# Relationship Between Serum Albumin and Risk of Atrial Fibrillation: A Dose-Response Meta-Analysis

**DOI:** 10.3389/fnut.2021.728353

**Published:** 2021-08-18

**Authors:** Yali Wang, Peng Du, Qi Xiao, Jianfeng Li, Xiao Liu, Jinfeng Tan, Xingjian Zhang

**Affiliations:** ^1^Department of Pathology, The Affiliated Stomatological Hospital of Nanchang University, Nanchang, China; ^2^Department of General Surgery, The First Affiliated Hospital of Nanchang University, Nanchang, China; ^3^Department of Cardiology, Sun Yat-sen Memorial Hospital, Sun Yat-sen University, Guangzhou, China; ^4^Guangdong Province Key Laboratory of Arrhythmia and Electrophysiology, Sun Yat-sen Memorial Hospital, Sun Yat-sen University, Guangzhou, China

**Keywords:** atrial fibrillation, dose-response, risk factor, nutrition, albumin

## Abstract

**Background:** The dose–response association between serum albumin and atrial fibrillation is not well known. This study aims to assess the relationship between albumin and atrial fibrillation and the potential dose–response effect.

**Methods:** Studies reported that the serum albumin and AF were identified by searching the EMBASE, PubMed, and Cochrane Library databases. The potential dose–response effect was performed by using a stage robust error meta-regression.

**Results:** Nine studies were included with a total of 32,130 individuals. Patients with high albumin level were associated with a decreased risk of atrial fibrillation compared with patients with low serum albumin (OR[odds ratio]: 0.62, 95% CI [0.44, 0.89]; *I*^2^ = 76%; *P* = 0.009). In the dose–response analysis, for each 10 g/L increase in serum albumin level, the risk of atrial fibrillation decreased by 36% (95% CI: 0.51–0.81, *I*^2^ = 87%, *P* < 0.001). Furthermore, a significant negative linear relationship between serum albumin and the risk of atrial fibrillation (*P*_nonlinearity_ = 0.33) was found.

**Conclusion:** Our dose–response meta-analysis suggests that low serum albumin level is associated with an increased risk of atrial fibrillation. Further studies are needed to explore the effect of induction of elevated serum albumin levels on the prevention of atrial fibrillation.

## Introduction

Atrial fibrillation (AF) is the most common arrhythmia in the clinical setting, with a substantially increased morbidity, mortality and bring tremendous economic. Although multiple risk factors, including cardiovascular and noncardiovascular risk factors, have been identified (1), the potential modifiable risk factors are yet to be explored.

Albumin is a predominant protein in human plasma (2). Hypoalbuminemia, usually defined as <35 g/L, is a significant indicator of malnutrition, inflammation, or cachexia and has been recognized as a robust biomarker for various noncardiovascular and cardiovascular diseases (3, 4) in the general population or patients with coexisting diverse comorbidities (5, 6). Several studies and our previous study have reported the malnutrition status and the risk of AF (7–11) as an indicator of malnutrition, however, the serum albumin and associated risk of AF is inconclusive. Moreover, the dose–response relationship of albumin and AF is still unclear. Given this background, we aim to perform a meta-analysis to assess the relationship between albumin and AF and the potential dose–response effect.

## Methods

We performed this meta-analysis following the Preferred Reporting Items for Systematic Reviews and Meta-Analyses (PRISMA) statement (Supplementary Table 1) (12).

### Search Strategy

Database search, selection, data extraction, and statistical analysis were independently conducted by two authors (YL and XL). For all relevant studies, three databases (PubMed, Embase, and the Cochrane Library) were searched up to May 27, 2021. No language restrictions were applied. The following search terms were used for all of the databases: (“albumin” OR “hypoalbuminemia” OR “serum albumin”) AND (“atrial fibrillation” OR “auricular fibrillation”). Report data on adults ≥18 years of age that helped assess the association between serum albumin and risk of AF were included. In addition, the conference abstracts and bibliographies of related works of literature were scanned. The detail of the search strategy was listed in Supplementary Table 2.

### Selection Criteria and Study Selection

Following studies that satisfied the inclusion criteria were selected: (1) studies that reported the serum albumin level and risk of AF; (2) those designed as randomized controlled trials or observational studies; (3) those that made available a quantitative measure of serum albumin level and the corresponding estimate effect and 95% CI in each albumin category for the dose–response analysis. Accordingly, reviews, meta-analyses, congress abstracts, practice guidelines, patents, cases, editorials, replies, or comments studies were excluded. Any inconsistency was resolved through discussions (YL and XL) until consensus was reached.

### Data Collection and Quality Assessment

Data of each study were extracted based on prespecified inclusion criteria, including the first author, publication year, geographical location, study type, participants (sex, age, and sample size), adjustments for confounders, categories of serum albumin, and adjusted odds ratios (ORs) with its 95% confidence intervals (CIs) for each serum albumin category. We included the articles with the longest follow-up or the largest numbers of participants for multiple publications and reports by using the same data. The Newcastle–Ottawa quality scale (NOS) or the Joanna Briggs Institute (JBI) critical appraisal checklist (cross-sectional study) was applied for quality assessment (13). The JBI critical appraisal checklist (https://jbi.global/critical-appraisal-tools) composed eight items, which score each study for the following items: participant selection, exposure definition, statistical analysis, and outcome data. Studies with a NOS or JBI of ≥6 stars were considered moderate- to high-quality articles (14).

### Statistical Analysis

Summary ORs and 95% CIs for a 10-g/L increment in serum albumin were pooled by using a random-effects model after considering the potential heterogeneity. The risk ratio is treated as equal to ORs (15). We calculated the study-specific slopes (linear trends) and 95% CIs from the natural logs of the reported ORs and CIs across categories of serum albumin by using the method described by Greenland and Longnecker (16). Nonlinear dose–response models were fitted by using the robust error meta-regression method (17, 18) that required at least two quantitative serum albumin level ORs with variance estimates. We estimated the midpoint of each category by averaging the lower and upper boundaries of that category if the median or mean serum albumin was not provided and reported in ranges (19). If the highest or lowest category was open-ended, we assumed that the open-ended interval length was the same as the adjacent interval (15). To assess the heterogeneity of ORs across studies, the *I*^2^ (95% CI) statistic was calculated with the following interpretation: low heterogeneity, defined as *I*^2^ < 50%; moderate heterogeneity, defined as *I*^2^ 50%−75%; and high heterogeneity, defined as *I*^2^ > 75% (15). Moreover, subgroup analyses were stratified by variables of interest, such as mean age, study population, study design, and adjustments. All analyses were performed using Stata 16.0 (Stata Corp LP, College Station, TX, USA) and Review Manager (RevMan) version 5.3 (The Cochrane Collaboration 2014; Nordic Cochrane Center Copenhagen, Denmark). A *P* < 0.05 was considered statistically significant.

## Results

### Literature Search

**Figure 1:**
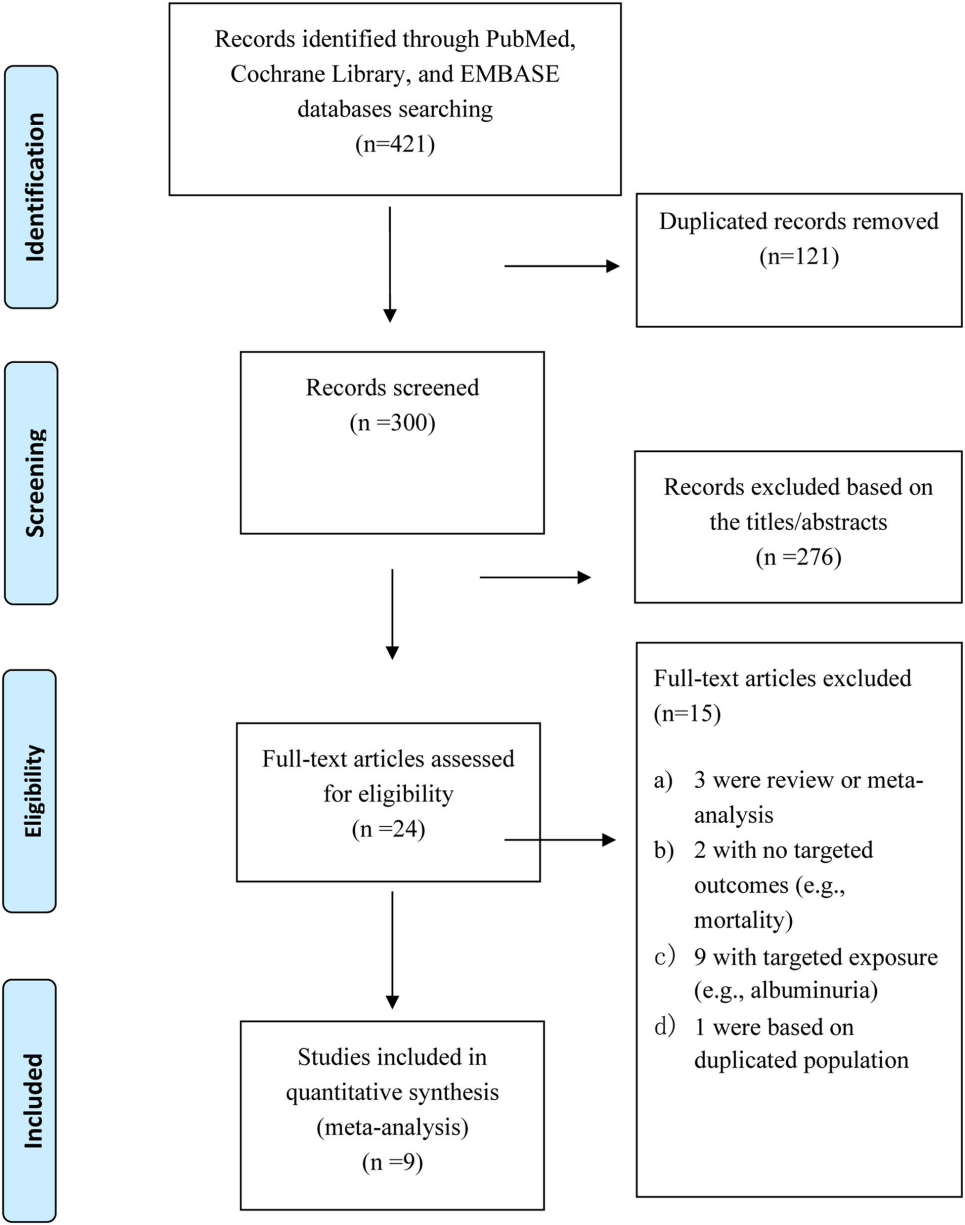
Flow chart of this meta-analysis.

The selection process of the study is shown in Figure 1. We identified 421 articles through an initial database search. After removing the duplicated articles, 300 reports remained. We further excluded 276 articles by quickly screening the titles and abstracts and reviewed 24 articles in more detail. Of the 24 records, 15 were excluded (e.g., reviews and duplicated population) after the full-text review. The details of the reasons to exclude them are described in Supplementary Table 3. Finally, 9 (7–11, 20–23) eligible studies (10 cohorts) with 32,130 individuals were included.

### Study Characteristics and Study Quality

The baseline characteristics of each study were listed in Table 1. Overall, six studies reposted a positive association between the serum albumin and risk of AF, while three cohorts showed a null association. They were published from 2009 to 2020, with three from Europe, four from Asia, and two from United States. The mean age of participants ranged from 52 to 67 years old. AF diagnosis was based on the electrocardiogram among all works of literature. Four studies were prospective cohort, four were retrospective cohort, and one was a cross-sectional study. Two studies were general population based and others included hospitalized patients.

**Table 1:** Baseline characteristic of included studies in the association between serum albumin and atrial fibrillation.

**Author, year, Country**	**Sample size**	**Data source**	**Population**	**Design**	**Mean age (years), Male (%)**	**AF diagnosis/ Follow-up**	**Albumin level reported**	**OR**	**95%CI**	**Adjustments**	Study quality^*^
Beek (10), Netherlands	97	Maxima Medical Center	Hospitalized in ICU	Prospective cohort	67, 53	ECG/na	Per 1 g/l increase	0.86	0.77–0.97	Age, previous AF, prior use of anti-arrhythmic drug, severe sepsis, potassium, magnesium, calcium	6
Acar (11), Turkey	183	Karimnagar's State Hospital	Hemodialysis patients	Retrospective cohort	52, 48	ECG/na	Per 1 g/dl decrease	1.25	0.22–7.14	Age, right atrium diameter, left atrium diameter, Left ventricle mass index, E/E, pulmonary artery pressure, valvular calcification, duration of hemodialysis	6
Tanaka (9), Japan	1,524	Aichi Cohort Study	Chronic kidney disease	Prospective cohort	67, 68.5	ECG/na	Per 1 mg/dl increase	0.56	0.32–0.96	Age, sex, CAD, CRP	8
Liu (21), China	3,489	People's Hospital of Peking University	Inpatient	Retrospective cohort	62, 55.8	ECG/na	<35 g/L ≥35 g/L	2.02 Ref	1.43–2.84	Age, sex, heart failure, cardiomyopathy, rheumatic heart disease, hyperthyroidism, SUA	6
Mukamal (22), Danish	8,870	Copenhagen City Heart Study	Free of cardiovascular disease	Prospective cohort	55, na	ICD/7.5 years	women <461 g/L	Ref		Age, smoking, BMI, and sex, height, physical activity, education, family history of CVD, co-habitation, FEV1, systolic blood pressure, and history of diabetes	7
							461–478	2.04	1.10–3.80		
							478–496	1.10	0.57–2.13		
							>496	2.14	1.15–3.96		
							Men <468	Ref			
							468–486	1.41	0.64–3.09		
							486–506	1.41	0.64–3.09		
							>506	1.98	0.94–4.17		
Liao (8), US	15,792	Atherosclerosis Risk in Communities	General Population	Prospective cohort	53, 54	ECG/25 years	Per 1 g/dl increase	0.67	0.56–0.80	Age, race, gender, BMI, waist-hip ratio, heart rate, smoking, drinking, education, income, heart failure, CAD, DM, hypertension, Na, K, creatinine, non-albumin protein, uric acid, HDL-c, LDL-c, triglycerides, total cholesterol, glucose, antihypertension medicine, stain, aspirin, anticoagulation medicine, white blood cell	8
							≤ 3.7 g/dl	Ref			
							3.7–3.9	0.87	0.78–0.97		
							3.9–4.0	0.83	0.72–0.95		
							> 4.0	0.80	0.71–0.91		
Karabacak (7), Turkey	830	University of Health Sciences	CABG	Retrospective cohort	63, 75	ECG/na	Per 1 g/dl increase	0.438	0.25-0.86	Age, CRP, CRP/albumin ratio	6
Mwalitsa (23), Italy	335	Liver Unit of the University Hospital of Messina”	Cirrhosis	Retrospective cohort	65, 65	ECG	Per 1 g/dl increase	0.41	0.05-0.33	None	5
Ananthapanyasut (20), US		Advocate Christ Medical Center	Non-dialysis Patients with Chronic Kidney Disease	Cross sectional	65, 52	ECG	Per 1 g/dl increase	0.48	0.39-0.58	None	6

*AF, atrial fibrillation; HF, heart failure; CRP, C-reactive protein; BMI, body mass index; DM, diabetes mellitus; eGFR, estimated glomerular filtration rate; HDL-C, high-density lipoprotein cholesterol; LDL-C, low-density lipoprotein cholesterol; ECG, Electrocardiography; ICD, International classification of diseases; SUA, serum uric acid; CVD, cardiovascular diseases; ICU, intensive care unit; FEV1, Forced Expiratory Volume in the first second; OR, odd ratio; CABG, coronary artery bypass graft; na, not available*.

* *Study quality was assessed by Newcastle-Ottawa Scale (cohort and case-control study) or Joanna Briggs Institute Critical Appraisal Checklist (cross-sectional study)*.

### Association Between Serum Albumin Level and Risk of AF

**Figure 2:**
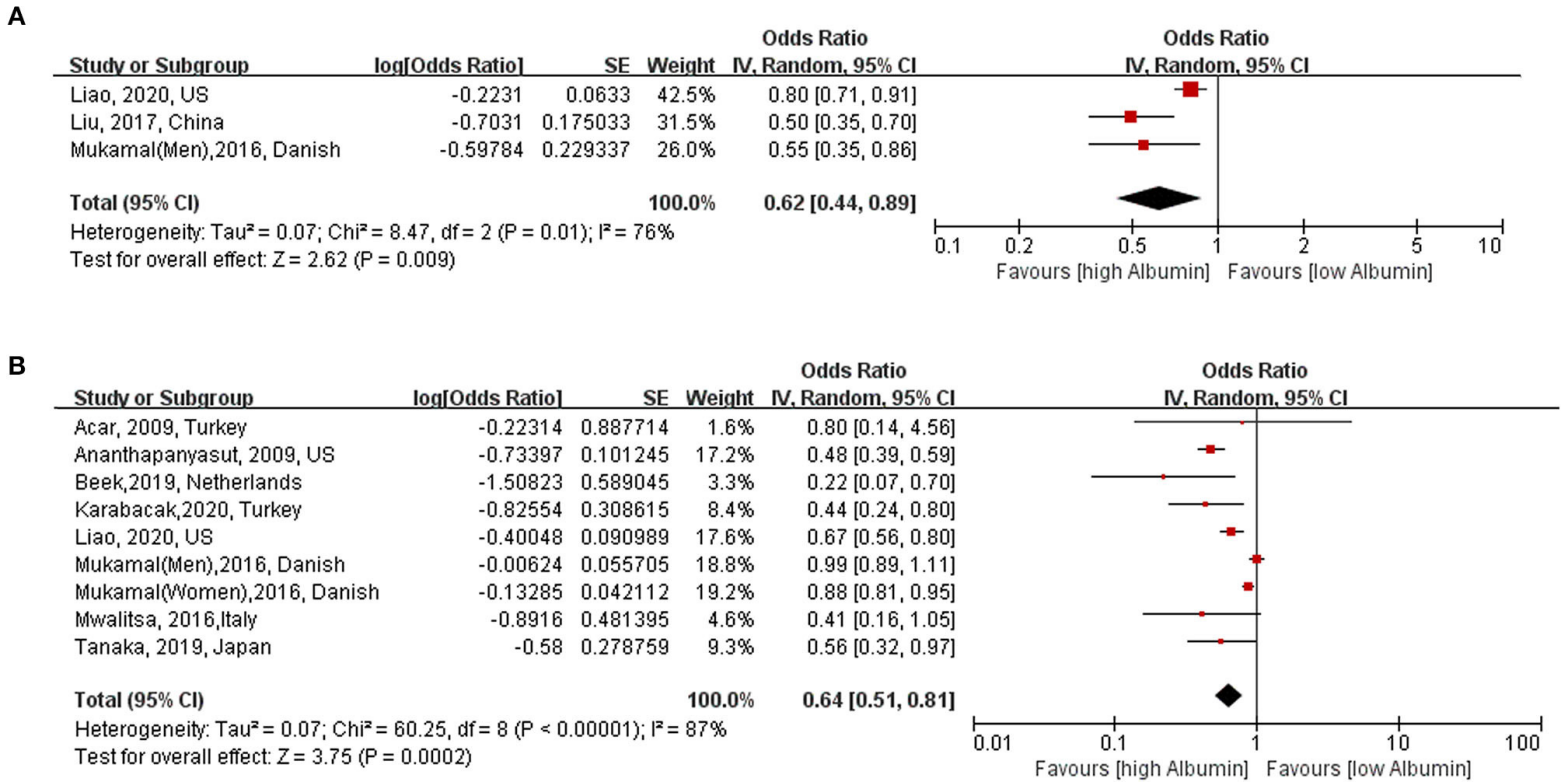
Association between serum albumin level and risk of atrial fibrillation. **(A)** Category analysis (highest vs. lowest) **(B**): Continuous analysis (per 10 g/L increase). AF, atrial fibrillation; OR, odd risk.

Three studies (8, 21, 22) with 28,651 reported category analyses between serum albumin and risk of AF. As shown in Figure 2A, high serum albumin level was associated with decreased risk of AF (OR: 0.62, 95% CI [0.44, 0.89]; *I*^2^ = 76%; *P* = 0.009) (highest vs. lowest) with significant heterogeneity.

**Figure 3:**
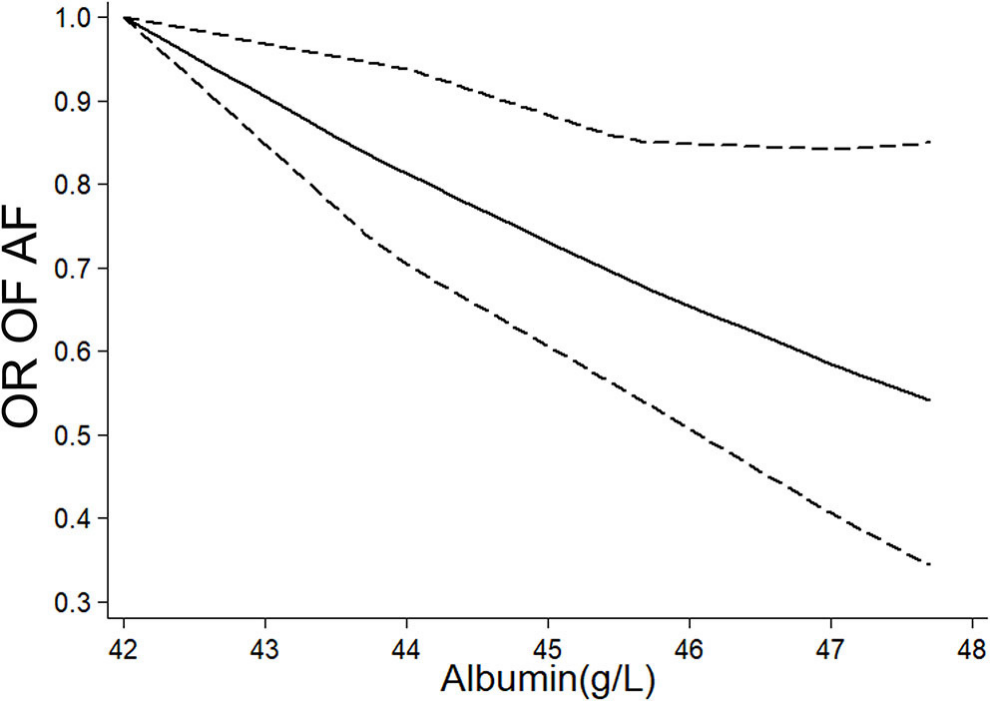
Nonlinear exposure–effect analysis between serum albumin level and risk of atrial fibrillation, the solid and dashed lines represent the estimated odd risk and the 95% confidence interval, respectively. AF, atrial fibrillation; OR, odd risk.

Eight studies (7–11, 20–23) (nine cohorts) with 28,641 participants were included in the exposure–effect analysis. A 10-g/L increment in serum albumin decreased the risk of AF by 36% (95% CI: 0.51–0.81, *I*^2^ = 87%, *P* < 0.001) (Figure 2B). Heterogeneity was not significant when excluding Liao et al. (8) while the summary OR was still significant (OR: 0.47, 95% CI: 0.40–0.56, *I*^2^ = 0%, *P* < 0.001). In the nonlinear model, there was a significant negative linear association between serum albumin and risk AF (*P*_linearity_ = 0.33) (Figure 3).

### Subgroup Analysis and Sensitive Analysis

**Figure 4:**
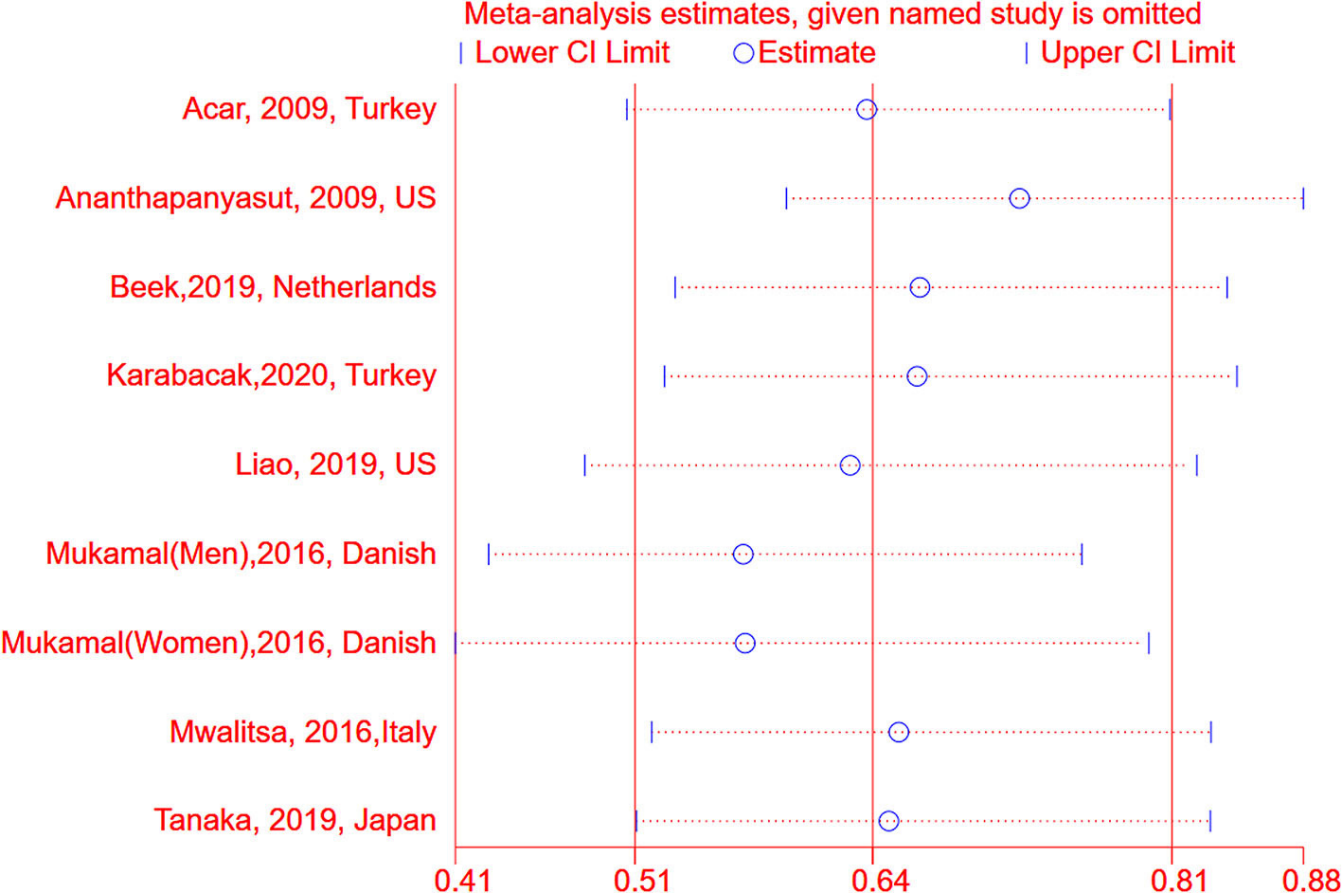
Sensitive analysis between serum albumin and risk of atrial fibrillation by omitting one study at each time. AF, atrial fibrillation; OR, odd risk.

We conducted subgroup analyses stratified by age, region, confounding factors, and potential intermediate factors. As shown in Table 2, among hospitalized patients, there was a significant inverse association between albumin and AF (OR: 0.48, 95% CI: 0.40–0.56, *I*^2^ = 0%, *P* < 0.001), however, it was not significant in the group of general population (OR: 0.85, 95% CI: 0.71–1.01, *I*^2^ = 56%, *P* = 0.07). The association between serum albumin and the risk of AF persisted in other subgroups, including age, regions, publication years, sample size or adjusted for age, sex, cardiovascular diseases, and C-reactive protein (Table 2). The results were stable by omitting one study at once (Figure 4), excluding the studies with crude analysis and those that reported AF postoperation or cross-sectional study (Table 2). According to the guidelines, meta-regression and publication bias were not conducted after considering the limited numbers of included studies (*N* <10).

**Table 2:** Subgroup analysis and sensitive analysis of serum albumin and risk of atrial fibrillation.

**Items**		**Number of cohorts**	**OR**	**I^**2**^%**	***P***
					**Within subgroup**
**Subgroup analysis**					
Mean age	<65 years	4	0.48 [0.40, 0.57]	58	<0.001
	≥65 years	5	0.81 [0.67, 0.97]	34	0.004
Region	Northern America	4	0.74 [0.57, 0.95]	94	0.02
	Europe	2	0.32 [0.15, 0.67]	0	0.002
	Asia	3	0.51 [0.35, 0.76]	92	0.002
Study quality	≤ 6 scores	3	0.47 [0.39, 0.57]	26	0.007
	6 high scores	6	0.78 [0.65, 0.94]	73	<0.001
Publication yeas	2009–2017	4	0.55 [0.39, 0.76]	38	0.003
	2017–2021	5	0.73 [0.54, 0.97]	64	0.01
Sample size	<1,000	6	0.40 [0.26, 0.64]	14	<0.001
	≥ 1,000	4	0.72 [0.56, 0.91]	92	0.007
Population	General population	3	0.85 [0.71, 1.01]	56	0.07
	Hospitalized patients	6	0.48 [0.40, 0.56]	0	<0.001
Adjusted for age	Yes	7	0.75 [0.61, 0.91]	78	0.003
	No	2	0.48 [0.39, 0.58]	21	<0.001
Study design	Prospective cohort	5	0.87 [0.82, 0.93]	82	<0.001
	Retrospective cohort	3	0.45 [0.28, 0.74]	0	0.001
	Others	1	0.48 [0.39, 0.59]	-	<0.001
Adjusted for sex	Yes	4	0.82 [0.69, 0.98]	82	0.03
	No	5	0.47 [0.39, 0.56]	0	<0.001
Adjusted for CAD	Yes	4	0.82 [0.69, 0.98]	82	0.03
	No	5	0.47 [0.39, 0.56]	0	<0.001
Adjusted for CRP	Yes	4	0.82 [0.69, 0.98]	82	0.03
	No	5	0.47 [0.39, 0.56]	0	<0.001
**Sensitivity analysis**					
	Excluding crude analysis	7	0.75 [0.61, 0.91]	78	0.003
	Excluding POAF	8	0.67 [0.53, 0.85]	88	0.001
	Excluding cross-sectional studies	8	0.73 [0.60, 0.88]	76	0.001

*CRP, C-reactive protein; CVD, cardiovascular diseases; POAF, post-operation atrial fibrillation; OR, odd ratio*.

## Discussion

This study showed a significant negative linear association between serum albumin and risk of AF, where 10-g/L serum albumin decreased the risk of AF by 36%. The results were stable in most of the subgroup and sensitive analysis, suggesting the robustness of our findings. To the best of our knowledge, this is the first meta-analysis to assess serum albumin and AF association.

Atrial fibrillation occurs in fewer than 1% of persons aged 60–65 years; however, it could reach 8–10% of those older than 80 years (1). A firm link between hypoalbuminemia and the incidence of many cardiovascular diseases was established, such as ischemic diseases, stroke, and diabetes (24–27). Moreover, these associations were independent of conventional risk factors, such as body mass index, liver function, and inflammation. Consistently, in the present study, an inverse association between serum albumin level and the risk of AF was found. There Several potential mechanisms have been prosposed. Inflammation has been identified as one of the important mechanisms contributing to the incidence of AF (28). Serum albumin exerts a powerful anti-inflammatory function in physiological conditions (29). Several studies have reported that albumin has a positive association with various circulating levels of inflammatory factors, such as tumor necrosis factor-alpha and C-reactive protein (29). Furthermore, albumin represents a very abundant and important circulating antioxidant (e.g., free radicals and reactive oxygen species) (29, 30). Free-radical-associated damage is an important factor in the pathological processes of AF (31). Experimental studies showed that elevated reactive oxygen species might modify ion channel activity to increase AF susceptibility by increasing sarcoplasmic reticulum Ca^2+^ release *via* enhanced ryanodine receptor activity, shortening of the atrial action potential duration, and producing delayed afterdepolarizations (31, 32).

Our subgroup results showed a significant association between albumin and AF in the general population; however, it was not significant in patients with comorbidities (hospitalized patients). There might be several potential expansions. First, most of the studies based on hospitalized patients with comorbidities were retrospective and might introduce a larger bias. The residue confounding might enlarge the association of albumin and AF. Second, recent studies also have shown that albumin was a potential connection with cardiovascular diseases and diabetes. Therefore, we propose that the association between low serum albumin and AF may be amplified in patients with comorbidities or at high risk of developing comorbidities. However, considering the limited sample size, the role of serum albumin in patients with comorbidities or at high comorbidities risk needs to be further investigated. Further studies with prospective design and a larger sample size are required.

Previous findings showed discrepant effects of albumin among men and women. Among women of the Copenhagen City Heart Study, a protective effect was noted with a significant linear association. All categories of albumin showed no benefit of AF compared with lowest ablumin level among men. In contrast, findings from the Atherosclerosis Risk in Communities cohort showed a significant protective effect of albumin, regardless of sex. The contrasting results regarding sex might derive from different baseline characteristics, such as region, race, age, and follow-up time. For example, the incidence of AF in the Copenhagen City Heart Study and Atherosclerosis Risk in Communities cohort differ significantly, with 3.2% (286/8864, 7.5 years follow-up) for the Copenhagen City Heart Study and 17.6% (2259/12833, 25.1 years follow-up) for the Atherosclerosis Risk in Communities cohort. The protective effect of albumin among men might appear as the prolonged time of follow-up in the Copenhagen City Heart Study. Therefore, it is still unclear whether there is a gender difference in the association between albumin and AF and should be further studied.

## Clinical Implications

The measurement of serum albumin is simple, cheap, and routinely available. Given the potential link between low serum albumin level and AF, albumin supplement (such as albumin infusion or protein supplementation) for patients with hypoalbuminemia might be a therapeutic target for reducing the risk of AF. Decreased serum albumin levels imply an undernourished state, which usually leads to poor clinical outcomes. For example, AF post cardiac operation was one of the most common complications, low albumin levels have been reported to be a significant predictor for AF after coronary artery bypass graft surgery (7). However, currently, there is limited evidence to assess the effect of albumin on reducing the risk of AF. Further studies are needed to identify the role of albumin in the prevention of AF.

## Strengths And Limitation

This is the first meta-analysis that reported the association between albumin and AF risk and the robustness of the findings in multiple subgroup analyses. However, several limitations also should be recognized. First, this is an observation-based study, which cannot deduce the causal relationship. Low serum albumin level is an indicator of undernutrition status. Serum albumin might be an intermediate factor in the association between undernutrition status and risk of AF. Unmeasured and insufficiently measured variables could have resulted in residual confounding. Second, as we know, sex is an independent risk factor for AF. A sex difference might exist in the association between albumin and the risk of AF (22). However, we could also not perform a subgroup analysis stratified by sex due to data restriction. Third, the number of included studies is limited. Further larger and well-designed studies were needed to confirm our conclusion. Fourth, the protocol of this study was not prospectively registered, however, no similar protocols of this topic were registered in the International prospective register of systematic reviews (PROSPERO) database (https://www.crd.york.ac.uk/prospero/). Finally, there was substantial heterogeneity in the primary analysis, which may derive from the baseline characteristics of patients. However, there was no evidence of heterogeneity when Liao et al. was excluded, with results remaining positive.

## Conclusion

Our dose–response suggested low serum albumin level is significantly associated with an increased risk of AF. Further studies are needed to explore the effect of induction of elevated serum albumin levels on the prevention of AF.

## Data Availability Statement

The original contributions presented in the study are included in the article/Supplementary Material, further inquiries can be directed to the corresponding author/s.

## Author Contributions

XZ and JT were responsible for the entire project and revised the draft. XL and YW performed the systematic literature review and drafted the first version of the manuscript. All authors took part in the interpretation of the results and prepared the final version of the manuscript.

## Conflict of Interest

The authors declare that the research was conducted in the absence of any commercial or financial relationships that could be construed as a potential conflict of interest.

## Publisher's Note

All claims expressed in this article are solely those of the authors and do not necessarily represent those of their affiliated organizations, or those of the publisher, the editors and the reviewers. Any product that may be evaluated in this article, or claim that may be made by its manufacturer, is not guaranteed or endorsed by the publisher.

## Abbreviations

AF: Atrial fibrillation
CI: Confidence interval
OR: Odds ratio
NOS: Newcastle-Ottawa Scale score.
