# Supplementary material for: Relationship Between Serum Albumin and Risk of Atrial Fibrillation: A Dose-Response Meta-Analysis
Source: Front Nutr. 2021 Aug 18;8:728353. doi: 10.3389/fnut.2021.728353 (PMC8418186; doi:10.3389/fnut.2021.728353)
Supplement: Supplementary file 1 [file Data_Sheet_1.pdf]

PRISMA 2021 Checklist

Relationship between serum albumin and risk of atrial fibrillation: A dose-response meta-analysis

SUPPLEMENTAL MATERIALS

Table S1: Preferred Reporting Items for Systematic Review and Meta-Analyses (PRISMA) guidelines

Section/topic	#	Checklist item	Reported on page #
**TITLE**			
Title	1	Identify the report as a systematic review, meta-analysis, or both.	1
**ABSTRACT**			
Structured summary	2	Provide a structured summary including, as applicable: background; objectives; data sources; study eligibility criteria, participants, and interventions; study appraisal and synthesis methods; results; limitations; conclusions and implications of key findings; systematic review registration number.	2
**INTRODUCTION**			
Rationale	3	Describe the rationale for the review in the context of what is already known.	2
Objectives	4	Provide an explicit statement of questions being addressed with reference to participants, interventions, comparisons, outcomes, and study design (PICOS).	2
**METHODS**			
Protocol and registration	5	Indicate if a review protocol exists, if and where it can be accessed (e.g., Web address), and, if available, provide registration information including registration number.	Not applicable
Eligibility criteria	6	Specify study characteristics (e.g., PICOS, length of follow-up) and report characteristics (e.g., years considered, language, publication status) used as criteria for eligibility, giving rationale.	3
Information sources	7	Describe all information sources (e.g., databases with dates of coverage, contact with study authors to identify additional studies) in the search and date last searched.	2

PRISMA 2021 Checklist

Search	8	Present full electronic search strategy for at least one database, including any limits used, such that it could be repeated.	3
Study selection	9	State the process for selecting studies (i.e., screening, eligibility, included in systematic review, and, if applicable, included in the meta-analysis).	3
Data collection process	10	Describe method of data extraction from reports (e.g., piloted forms, independently, in duplicate) and any processes for obtaining and confirming data from investigators.	4
Data items	11	List and define all variables for which data were sought (e.g., PICOS, funding sources) and any assumptions and simplifications made.	4
Risk of bias in individual studies	12	Describe methods used for assessing risk of bias of individual studies (including specification of whether this was done at the study or outcome level), and how this information is to be used in any data synthesis.	4
Summary measures	13	State the principal summary measures (e.g., risk ratio, difference in means).	2
Synthesis of results	14	Describe the methods of handling data and combining results of studies, if done, including measures of consistency (e.g., I^2) for each meta-analysis.	3
Risk of bias across studies	15	Specify any assessment of risk of bias that may affect the cumulative evidence (e.g., publication bias, selective reporting within studies).	3
Additional analyses	16	Describe methods of additional analyses (e.g., sensitivity or subgroup analyses, meta-regression), if done, indicating which were pre-specified.	Not performed
**RESULTS**			
Study selection	17	Give numbers of studies screened, assessed for eligibility, and included in the review, with reasons for exclusions at each stage, ideally with a flow diagram.	4
Study characteristics	18	For each study, present characteristics for which data were extracted (e.g., study size, PICOS, follow-up	4

		period) and provide the citations.	
Risk of bias within studies	19	Present data on risk of bias of each study and, if available, any outcome level assessment (see item 12).	4
Results of individual studies	20	For all outcomes considered (benefits or harms), present, for each study: (a) simple summary data for each intervention group (b) effect estimates and confidence intervals, ideally with a forest plot.	4
Synthesis of results	21	Present results of each meta-analysis done, including confidence intervals and measures of consistency.	5
Risk of bias across studies	22	Present results of any assessment of risk of bias across studies (see Item 15).	5
Additional analysis	23	Give results of additional analyses, if done (e.g., sensitivity or subgroup analyses, meta-regression [see Item 16]).	5-6
**DISCUSSION**			
Summary of evidence	24	Summarize the main findings including the strength of evidence for each main outcome; consider their relevance to key groups (e.g., healthcare providers, users, and policy makers).	7
Limitations	25	Discuss limitations at study and outcome level (e.g., risk of bias), and at review-level (e.g., incomplete retrieval of identified research, reporting bias).	7
Conclusions	26	Provide a general interpretation of the results in the context of other evidence, and implications for future research.	8
**FUNDING**			
Funding	27	Describe sources of funding for the systematic review and other support (e.g., supply of data); role of funders for the systematic review.	8

Table S2: Detailed description of the search strategy

search strategy	
#1	albumin
#2	hypoalbuminemia
#3	serum albumin
#4	atrial fibrillation
#5	auricular fibrillation
#6	#1 OR #2 OR # 3
#7	#4 OR #5
#8	#6 AND #7

Table S3: Studies excluded with reasons

Studies excluded	Reasons
Alonso et al.[1]	Not the target exposure: albuminuria
Arques S et al.[2]	Review
Asselbergs et al.[3]	Not the target exposure: albuminuria
Baber et al.[4]	Not the target exposure: albuminuria
Bansal et al.[5]	Not the target exposure: albuminuria
Molnar et al.[6]	Not the target exposure: albuminuria
To et al, 2007[7]	Not the target exposure: albuminuria
Watanabe et al.[8]	Not the target exposure: albuminuria
Wetterslev et al.[9]	Meta-analysis
Xia et al.[10]	Meta-analysis
Zanetti et al.,[11]	Not the target exposure: albuminuria
Zhu et al.[12]	Not the target exposure: albuminuria
Kawai et al.[13]	Not the target outcome: mortality in atrial fibrillation patients
Dziedzic et al.[14]	Not the target outcome: ischemic stroke outcome
Misialek et al.[15]	Duplicated population (ARIC cohort)

1. Alonso A, Lopez FL, Matsushita K, Loehr LR, Agarwal SK, Chen LY, Soliman EZ, Astor BC and Coresh J. Chronic kidney disease is associated with the incidence of atrial fibrillation: the Atherosclerosis Risk in Communities (ARIC) study. *Circulation*. 2011;123:2946-53.
2. Arques S. Human serum albumin in cardiovascular diseases. *Eur J Intern Med*. 2018;52:8-12.
3. Asselbergs FW, van den Berg MP, Diercks GF, van Gilst WH and van Veldhuisen DJ. C-reactive protein and microalbuminuria are associated with atrial fibrillation. *Int J Cardiol*. 2005;98:73-7.
4. Baber U, Howard VJ, Halperin JL, Soliman EZ, Zhang X, McClellan W, Warnock DG and Muntner P. Association of chronic kidney disease with atrial fibrillation among adults in the United States: REasons for Geographic and Racial Differences in Stroke (REGARDS) Study. *Circ Arrhythm Electrophysiol*. 2011;4:26-32.
5. Bansal N, Zelnick LR, Alonso A, Benjamin EJ, de Boer IH, Deo R, Katz R, Kestenbaum B, Mathew J, Robinson-Cohen C, Sarnak MJ, Shlipak MG, Sotoodehnia N, Young B and Heckbert SR. eGFR and Albuminuria in Relation to Risk of Incident Atrial Fibrillation: A Meta-Analysis of the Jackson Heart Study, the Multi-Ethnic

Study of Atherosclerosis, and the Cardiovascular Health Study. *Clin J Am Soc Nephrol*. 2017;12:1386-1398.
6. Molnar AO, Eddeen AB, Ducharme R, Garg AX, Harel Z, McCallum MK, Perl J, Wald R, Zimmerman D and Sood MM. Association of Proteinuria and Incident Atrial Fibrillation in Patients With Intact and Reduced Kidney Function. *J Am Heart Assoc*. 2017;6.
7. To AC, Yehia M and Collins JF. Atrial fibrillation in haemodialysis patients: do the guidelines for anticoagulation apply? *Nephrology (Carlton)*. 2007;12:441-7.
8. Watanabe H, Watanabe T, Sasaki S, Nagai K, Roden DM and Aizawa Y. Close bidirectional relationship between chronic kidney disease and atrial fibrillation: the Niigata preventive medicine study. *Am Heart J*. 2009;158:629-36.
9. Wetterslev M, Haase N, Hassager C, Belley-Cote EP, McIntyre WF, An Y, Shen J, Cavalcanti AB, Zampieri FG, Guimaraes HP, Granholm A, Perner A and Moller MH. New-onset atrial fibrillation in adult critically ill patients: a scoping review. *Intensive care medicine*. 2019;45:928-938.
10. Xia M, Zhang C, Gu J, Chen J, Wang LC, Lu Y, Huang CY, He YM and Yang XJ. Impact of serum albumin levels on long-term all-cause, cardiovascular, and cardiac mortality in patients with first-onset acute myocardial infarction. *Clin Chim Acta*. 2018;477:89-93.
11. Zanetti D, Bergman H, Burgess S, Assimes TL, Bhalla V and Ingelsson E. Urinary Albumin, Sodium, and Potassium and Cardiovascular Outcomes in the UK Biobank: Observational and Mendelian Randomization Analyses. *Hypertension*. 2020;75:714-722.
12. Zhu L, Chen M and Lin X. Serum albumin level for prediction of all-cause mortality in acute coronary syndrome patients: a meta-analysis. *Biosci Rep*. 2020;40.
13. Kawai M, Harada M, Motoike Y, Koshikawa M, Ichikawa T, Watanabe E and Ozaki Y. Impact of serum albumin levels on supratherapeutic PT-INR control and bleeding risk in atrial fibrillation patients on warfarin: a prospective cohort study. *IJC Heart & Vasculature*. 2019;22:111-116.
14. Dziedzic T, Slowik A and Szczudlik A. Serum albumin level as a predictor of ischemic stroke outcome. *Stroke*. 2004;35:e156-e158.
15. Misialek JR, Bekwelem W, Chen LY, Loehr LR, Agarwal SK, Soliman EZ, Norby FL and Alonso A. Association of White Blood Cell Count and Differential with the Incidence of Atrial Fibrillation: The Atherosclerosis Risk in Communities (ARIC) Study. *PLoS One*. 2015;10:e0136219.

Table S4. Quality assessment of included studies

Author (Publication Year)	Newcastle-Ottawa Scale (NOS) or Joanna Briggs Institute Critical Appraisal Checklist (JBI)									
	Selection			Comparability			Outcome			Total
	a	b	c	d	e	f	g	h	i	
Beek,2019, Netherlands	1	1	1	1	1	1	1	0	1	6
Acar, 2009, Turkey	1	1	1	1	1	1	1	0	0	6
Tanaka, 2019, Japan	1	1	1	0	1	1	1	1	1	8
Liu, 2017 China	1	1	1	1	1	1	1	0	1	6
Mukamal,2016, Danish	1	1	1	0	1	1	1	1	0	7
Liao, 2019, Europe	1	1	1	1	1	1	1	1	1	8
Karabacak,2020, Turkey	1	1	1	1	1	1	1	1	1	6
Mwalitsa, 2016, Italy	1	1	1	0	1	1	1	1	0	5
Ananthapanyasut, US*	yes	yes	yes	yes	no	no	yes	yes	-	6

a. Representativeness of the exposed cohort.
b. Selection of the non-exposed cohort.
c. Ascertainment of exposure.
d. Demonstration that outcome of interest was not present at start of study.
e. Comparability of cohorts on the basis of the design or analysis (adjusted for age).
f. Comparability of cohorts on the basis of the design or analysis (adjusted for any other factor).
g. Assessment of outcome.
h. Was follow-up long enough for outcomes to occur.
i. Adequacy of follow-up of cohorts.

*Assessed by JBI checklist
